# Inter‐device agreement of sacral subepidermal oedema measurement in healthy adults during prolonged 60° head of bed elevation

**DOI:** 10.1002/nop2.2103

**Published:** 2024-01-31

**Authors:** Sharon L. Latimer, Madeline Bone, Rachel M. Walker, Lukman Thalib, Brigid M. Gillespie

**Affiliations:** ^1^ School of Nursing and Midwifery, Menzies Health Institute Queensland, NHMRC Centre of Research Excellence in Wiser Wounds Care Griffith University Southport Queensland Australia; ^2^ School of Nursing and Midwifery Griffith University Southport Queensland Australia; ^3^ School of Nursing and Midwifery, Menzies Health Institute Queensland, NHMRC Centre of Research Excellence in Wiser Wounds Care Griffith University Nathan Queensland Australia; ^4^ Metro South Health Brisbane Queensland Australia; ^5^ Department of Biostatistics, Faculty of Medicine Istanbul Aydin University Istanbul Turkey; ^6^ Gold Coast Hospital and Health Service Southport Queensland Australia

**Keywords:** pressure injuries, pressure injury risk, pressure ulcers, sub‐epidermal Oedema, wounds

## Abstract

**Aim:**

To investigate the level of agreement between the SEM 200 and Provisio® subepidermal moisture sacral delta measurements, which may indicate increased pressure injury risk, in healthy adults during 120 min of prolonged 60° head of bed elevation. This position, which requires the elevation of the patient's upper body at a 60° angle above the horizontal plane for an extended period, is used by clinicians to prevent or manage a patient's medical or surgical conditions.

**Design:**

This prospective exploratory study recruited 20 healthy adults during October 2021 and collected sacral subepidermal moisture delta measurements using the SEM 200 and Provisio® devices.

**Methods:**

Delta measurements were taken at 20‐min intervals over 120 min resulting in seven data collection timepoints. Descriptive statistics and a Bland Altman plot analysis were conducted.

**Results:**

A total of 280 sacral subepidermal moisture delta measurements were gathered or 140 per device. There were good levels of agreement between the two devices at baseline (T0) [mean 0.025; SD 0.137] and following 60‐ (T3) [mean 0.025; SD 0.111], 80‐ (T4) [mean −0.01; SD 0.177] and 100 min (T5) [mean 0.01; SD 0.129] of prolonged 60° head of bed elevation. Head of bed elevations can increase a patient's risk of sacral pressure injuries. In some countries, nurses have access to the SEM 200 and/or the Provisio® device, so our findings may increase nurses' confidence in the interchangeability of the device measurements, although further research is needed to confirm this. The SEM 200 and Provisio® subepidermal moisture scanners show promise in gathering similar objective pressure injury risk data which could prompt clinicians to implement prevention strategies.

**Impact:**

Current pressure injury risk assessment is largely subjective in nature. This quantitative study on healthy human sacral tissue found a good level of agreement in the SEM 200 and Provisio® subepidermal moisture scanners, which may increase nurses' confidence in the interchangeability of the devices in clinical practice.

## 1 INTRODUCTION

Pressure injuries (PI) are localised skin and tissue injuries caused by shear, friction or prolonged pressure ranging between 1 and 6 h (European Pressure Ulcer Advisory Panel et al., 2019; Gefen, 2008). These injuries can cause in an inflammatory response resulting in microvascular permeability and localised subepidermal oedema (Gefen, 2018). A cascade of events then results in cell death, tissue pH deviations, and additional subepidermal oedema (Gefen, 2018). If the pressure is not relieved, the first signs of a PI can emerge including localised skin redness (erythema) and heat (Gefen & Ross, 2020). For patients, PI can result in lifelong physical and emotional impacts (Latimer et al., 2014) while burdening clinicians with increased workloads (Latimer et al., 2021).

## 2 BACKGROUND

PIs are avoidable adverse events which some patients develop during their hospitalisation. A 2020 meta‐analysis of more than 680,000 patients examined the extent of PI in hospital, with the authors reporting a 12.8% (95% confidence intervals: 11.8–13.9) prevalence and a pooled incidence rate of 5.4/10,000 patient days (Li et al., 2020). Furthermore, PI most frequently developed on the sacrum (37.3%), heels (29.5%) and hips (7.8%) (Li et al., 2020). For hospital systems, treating and managing PI is costly, with annual estimates in the United Kingdom of GBP 1.4–2.1 billion (Bennett et al., 2004), USD 26.8 billion in the United States (Padula & Delarmente, 2019) and AUD 9.11 billion in Australia (Nghiem et al., 2022).

A patient's risk of PI development increases with factors such as advancing age, immobility and poor nutritional status (European Pressure Ulcer Advisory Panel et al., 2019). PI prevention clinical practice guidelines recommend a head of bed elevation (HOBE) ≤ 30°, which involves the elevation of the patient's upper body above the horizontal plane at the required angle, can reduce a patient's PI risk, although, to adequately prevent or manage some medical and surgical conditions, such as pneumonia, patients require higher elevations (Güner & Kutlutürkan, 2022). While HOBE has clinical benefits, when implemented for extended periods without reprieve, it is also known to raise the external pressure exerted on the patient's sacrum and heels and increase their subsequent risk of PI (European Pressure Ulcer Advisory Panel et al., 2019). PI risk assessment tools (e.g. Waterlow, Braden, Norton) and visual skin assessment underpin prevention (European Pressure Ulcer Advisory Panel et al., 2019). Yet the subjective nature of visual skin assessment and variations in inter‐rater reliability impact their validity in detecting the early signs of PI development (European Pressure Ulcer Advisory Panel et al., 2019).

Objective bedside technologies, such as subepidermal moisture (SEM; Scafide et al., 2020), measure localised subepidermal oedema and are recommended as an adjunct to PI risk assessment (European Pressure Ulcer Advisory Panel et al., 2019). These devices provide quantitative data on the biocapacitance or electrical properties (Peko & Gefen, 2019) of inflamed tissue and subepidermal oedema, a potential precursor to PI, which may indicate underlying damage (Gefen & Ross, 2020). Evidence is growing on the potential benefits of SEM devices in PI risk assessment and the detection of underlying tissue damage (Bryant et al., 2021; Chaboyer et al., 2022; Martins de Oliveira et al., 2022; Scafide et al., 2020) up to 8 days before the first signs of a PI are visible (Martins de Oliveira et al., 2022).

**FIGURE 1:**
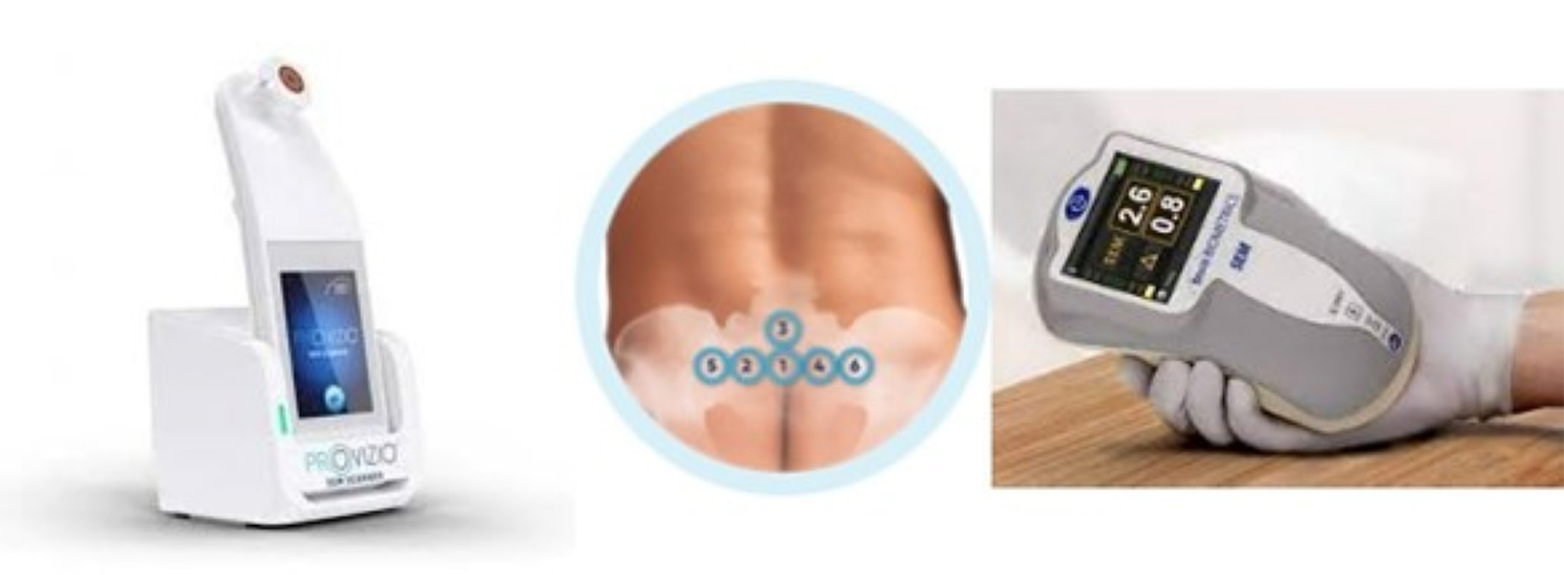
Provisio® SEM scanner, six sacral SEM absolute values, and SEM 200. Images used with permission from Bruin Biometrics 2022.

The SEM scanner devices (Figure 1) measure localised sacral subepidermal oedema by first obtaining six unitless SEM absolute values (SEM 200 range: 0.3–3.9; Provisio® range: 1.0–4.5; Bruin Biometrics, n.d.). Both devices then automatically display a SEM delta measurement, which is the difference between the highest and lowest of the six unitless SEM absolute values (Bruin Biometrics, n.d.). If the SEM delta is ≥0.6 this may suggest an increased PI risk, with a lower PI risk suggested if the SEM delta is <0.6 (Bruin Biometrics, n.d.; Bryant et al., 2021). Bruin Biometrics LLC introduced the first generation SEM 200 in 2013 with the second generation device, the Provisio®, released in 2019 (Bruin Biometrics, n.d.). Both devices have the same technology and are reported to perform identically in terms of their use and clinical interpretation (Bryant et al., 2021). Compared to the SEM 200, the Provisio® is more compact, user‐friendly and has integrated barcode capabilities (Bryant et al., 2021). To date, the published clinical SEM research features the first generation SEM 200 device (Gershon & Okonkwo, 2021; Martins de Oliveira et al., 2022). Although no clinical research papers using the second generation Provisio® were identified, Peko and Gefen (2020) recently conducted a laboratory test using this device. Ensuring the reliability and accuracy of the SEM 200 and Provisio® device is paramount to patient care and safety (Bryant et al., 2021) hence, rigorous clinical research is needed.

Only two studies have examined the inter‐device agreement between the SEM 200 and Provisio®. Good inter‐device agreement was found in the sacral, sternum and heel SEM measurements of 31 healthy volunteers using three SEM 200 devices (Clendenin et al., 2015). While, the laboratory studies conducted by Peko and Gefen's (Peko & Gefen, 2019, 2020) also found good levels of agreement between the SEM 200 and the Provisio® devices in identifying fluid changes following the injection of 1–4 mL of water into bioengineered phantom heel and facial cheek/chin tissue. Yet, little is known about the level of agreement between these two devices when sacral tissue is exposed to prolonged external pressure associated with HOBE; a known PI risk factor.

## 3 AIMS

The aim of this study was to assess the levels of agreement (Bland & Altman, 1999) in the sacral subepidermal oedema of healthy adults between the SEM 200 and Provisio® device during 120 min of prolonged 60° HOBE. This exploratory research will help to identify potential issues with clinical interpretation of the sacral SEM measurements during prolonged pressure loading and determine the interchangeability of both devices in clinical practice or if the second generation device (Provisio®) can replace the first generation device (SEM 200).

## 4 METHODS

### 4.1 Design

A prospective observational study was undertaken to determine the inter‐device agreement between the SEM 200 and Provisio® SEM scanner in measuring sacral subepidermal oedema in healthy adults during prolonged 60° HOBE.

The study was conducted in an airconditioned clinical nursing laboratory at a large university in southeast Queensland, Australia. Electric hospital beds fitted with a Prema Advanced III pressure reducing foam mattress were used to position participants supinely in a 60° HOBE for 120 min. Prior to recruitment and data collection, human research ethics committee clearance was obtained from the university (GU: 2021/515) and the study complied with the Australian national ethical standards.

### 4.2 Sampling and participants

We employed a convenience and snowball sampling approach. Our study population were healthy adult volunteers who worked or studied at the university and met the study inclusion criteria. The investigative nature of this study meant a small sample size of 20 participants was deemed sufficient (Bland & Altman, 1999; Leon et al., 2011). Campus‐wide study recruitment strategies (posters, newsletter advertisements and staff emails) were deployed in September 2021. Potential participants expressing an interest in recruitment received a verbal study overview, had their questions answered, and were informed participation was anonymous and voluntary. The study eligibility criteria included: aged ≥18 years, provide informed consent, and self‐reported good health. Exclusion criteria were sacral skin breaks, unable to lay supine in a 60° HOBE for 120 min, multiple medical comorbidities which increased their PI risk (e.g., peripheral vascular disease), and pregnancy. In total, 20 healthy adults meeting the study criteria were approached and all agreed to participate.

### 4.3 Instruments and training

In this study, the first generation SEM 200 and second generation Provisio® SEM scanners (Figure 1) were used. Both have United States FDA approval and are the only devices available on the market “intended for use as an adjunct to the standard of care when assessing the heels and sacrum of patients at increased risk for PIs” (Bryant et al., 2021, p. 834). Recent laboratory measurements of water variations in phantom models confirmed the sensitivity equivalence between the two devices (Peko & Gefen, 2020).

Using the SEM 200 and Provisio® SEM scanner devices as recommended by the manufacturer, at each data collection point six individual sacral readings were taken by gently pressing the device sensor against the skin in a pre‐determined order (Figure 1; Bruin Biometrics, n.d.). To increase standardisation of study processes, an operating procedure manual was developed and the research team completed 3 hours of training with a Bruin Biometrics representative (device operation, patient and skin preparation, operator consistency, documentation; Bruin Biometrics, n.d.).

### 4.4 Data collection

Over five consecutive days during October 2021, quantitative data were gathered from participants who also determined their preferred day and time of attendance in the clinical laboratory. Following written consent, self‐reported demographic data (sex, age, height and weight (used to calculate body mass index), smoking status, diabetes, peripheral vascular disease, ischaemic heart disease, hypertension, heart failure) were collected from participants. Using the SEM 200 and Provisio® SEM scanner, baseline (T0) sacral SEM absolute values and delta measurement were first gathered along with sacral skin inspection and a blanching test. Participants were then asked to lie supinely on a hospital bed at a 60° HOBE for 120 min. Every 20 min, participants were briefly repositioned laterally to facilitate collection of sacral SEM absolute values and delta measurements, using both devices (T1 [20 min], T2 [40 min], T3 [60 min], T4 [80 min], T5 [100 min], T6 [120 min]). This resulted in a total of seven data collection points (Table 1). Previous research found on average hospital patients repositioned themselves approximately 3.8 times per hour (Chaboyer et al., 2013), which informed our data collection schedule. At each data collection point, participants' sacral skin was inspected for erythema, a blanching test performed, and their sacral pain score (0–10) assessed. The gathered data were entered into a password protected Excel study‐specific spreadsheet.

**TABLE 1:** Paired *t*‐test: Sacral SEM delta difference (paired SEM 200 and Provisio®) during prolonged 60° HOBE (*n* = 20).

Timing of measurement	*t*	Df	*p* value (2 tailed)	Mean sacral SEM delta difference (SD)	95% CI of difference		Cohen's *d*
					Lower	Upper	
Baseline (T0)	0.815	19	0.425*	0.025 (0.137)	−0.039	0.089	0.182
20‐min (T1)	2.430	19	0.025	0.080 (0.147)	0.011	0.148	0.543
40‐min (T2)	2.666	19	0.015	0.070 (0.117)	0.015	0.125	0.596
60‐min (T3)	1.000	19	0.330*	0.025 (0.111)	−0.027	0.077	0.224
80‐min (T4)	−0.255	19	0.804*	−0.010 (0.177)	−0.093	0.073	−0.056
100‐min (T5)	0.346	19	0.733*	0.010 (0.129)	−0.050	0.070	0.077
120‐min (T6)	2.210	19	0.040	0.075 (0.151)	0.004	0.146	0.494

* Statistically non‐significant paired test.

### 4.5 Data analysis

Data were entered into the IBM Corp Statistical Package for Social Sciences (version 28.0) and its accuracy was double‐checked. There was no missing data. Descriptive statistics were computed based on the type of data (continuous/categorical) and distribution, reported as mean (standard deviation) or median (interquartile range) and frequencies (absolute [numbers] or proportions [%]). As the SEM 200 and Provisio® sacral SEM delta were continuous measures, the Bland–Altman plot was used to display the inter‐device agreement and paired *t*‐tests were used to test if the paired differences vary from a null value of zero (Bland & Altman, 1999; Clendenin et al., 2015).

**FIGURE 2:**
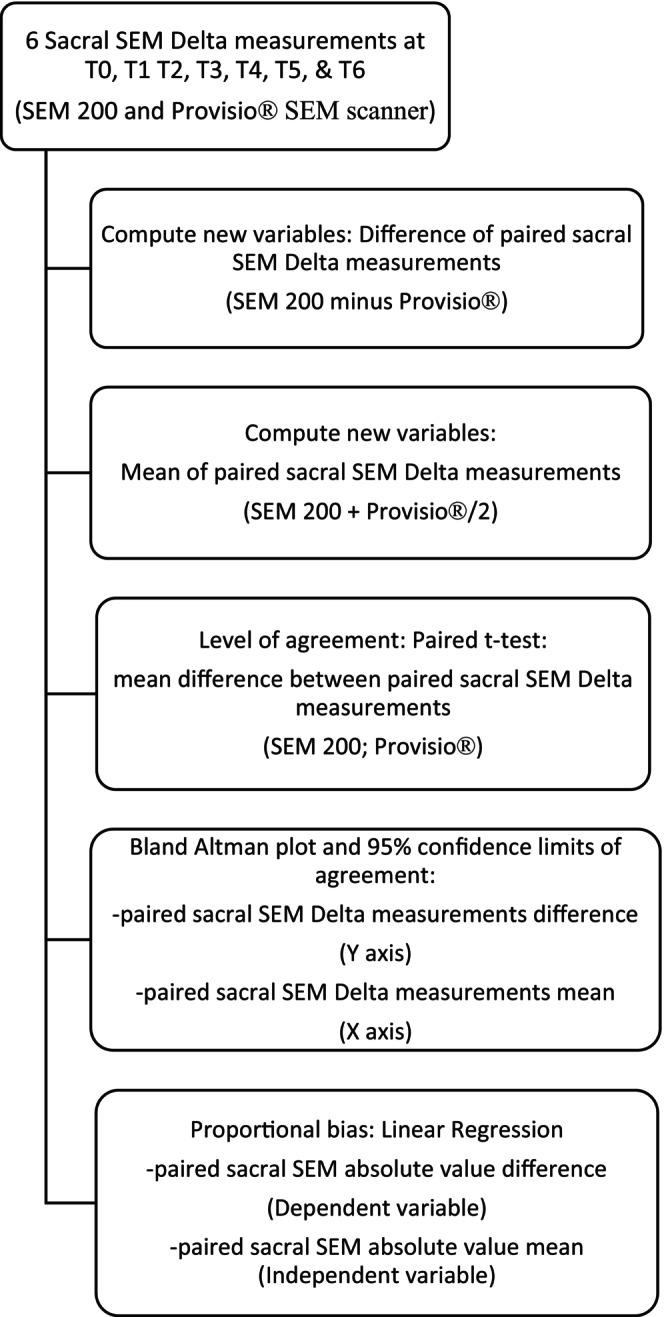
Bland Altman data analysis process: paired sacral SEM delta measurements.

The first step in the Bland–Altman analysis calculated the difference and mean between each pair of sacral SEM delta measurements gathered from T0 to T6 (Figure 2). Next, paired *t*‐tests were used to test the difference of the paired sacral SEM delta measurements to determine the level of agreement. We then used the Bland Altman plots at each time point to visually display the level of agreement (Bland & Altman, 1999). The Y‐axis variable, representing the difference between the paired sacral SEM delta measurements, was graphed against the mean of the sacral SEM delta measurements (X axis). The estimates were presented with 95% confidence limits of agreement (i.e. the mean difference ± [1.96 × standard deviation of the observed differences]; Bland & Altman, 1999). When interpreting the Bland Altman plot, a mean difference of zero indicates perfect agreement (Bland & Altman, 1999; Clendenin et al., 2015) between the devices. Finally, to determine proportional bias, a linear regression analysis was performed on the difference of the paired sacral SEM delta measurements (DV) and the mean of the paired sacral SEM delta measurements (IV). A *t* value close to zero coupled with a statistically non‐significant result meant no proportional bias was assumed or there were limited differences between the sacral measurements obtained by the SEM 200 and Provisio® SEM scanner.

## 5 RESULTS

The 20 healthy study participants were non‐smokers and aged between 24 to 67 years (Mean 39.3 years; SD 14.7) with slightly more males (*n* = 11; 55%) recruited. Their average body mass index (BMI) score was 25.8 (SD 4.3, 95% CI: 23.8–27.9) with slightly more than two‐thirds (*n* = 13; 65%) classified as overweight or obese (BMI ≥ 25.0). None of the participants self‐reported comorbidities of diabetes, peripheral vascular disease, ischaemic heart disease, hypertension or heart failure. In total, 1680 sacral SEM absolute values, 840 per device, were collected across the seven timepoints. This resulted in a total of 280 sacral SEM delta measurements or 140 per device. The sacral SEM absolute values for the SEM 200 ranged from 1.7 to 3.3 (Mean 2.6; SD 0.32) while the Provisio® range was 1.4–2.9 (Mean 2.3; SD 0.28). The sacral SEM delta measurements ranged from 0.1 to 0.8 for both devices, with a mean of 0.4 (SD 0.15) for the SEM 200 and 0.4 (SD 0.14) for the Provisio® device.

Table 1 reports the paired t‐test of the paired sacral SEM delta difference between the SEM 200 and the Provisio® at each 20‐min data collection point. Only 4 of the 7 (57.1%) paired‐tests were statistically non‐significant (T0, T3‐5). This demonstrates a level of agreement between the two devices at these timepoints. These results were used in the subsequent Bland Altman plot analysis.

**FIGURE 3:**
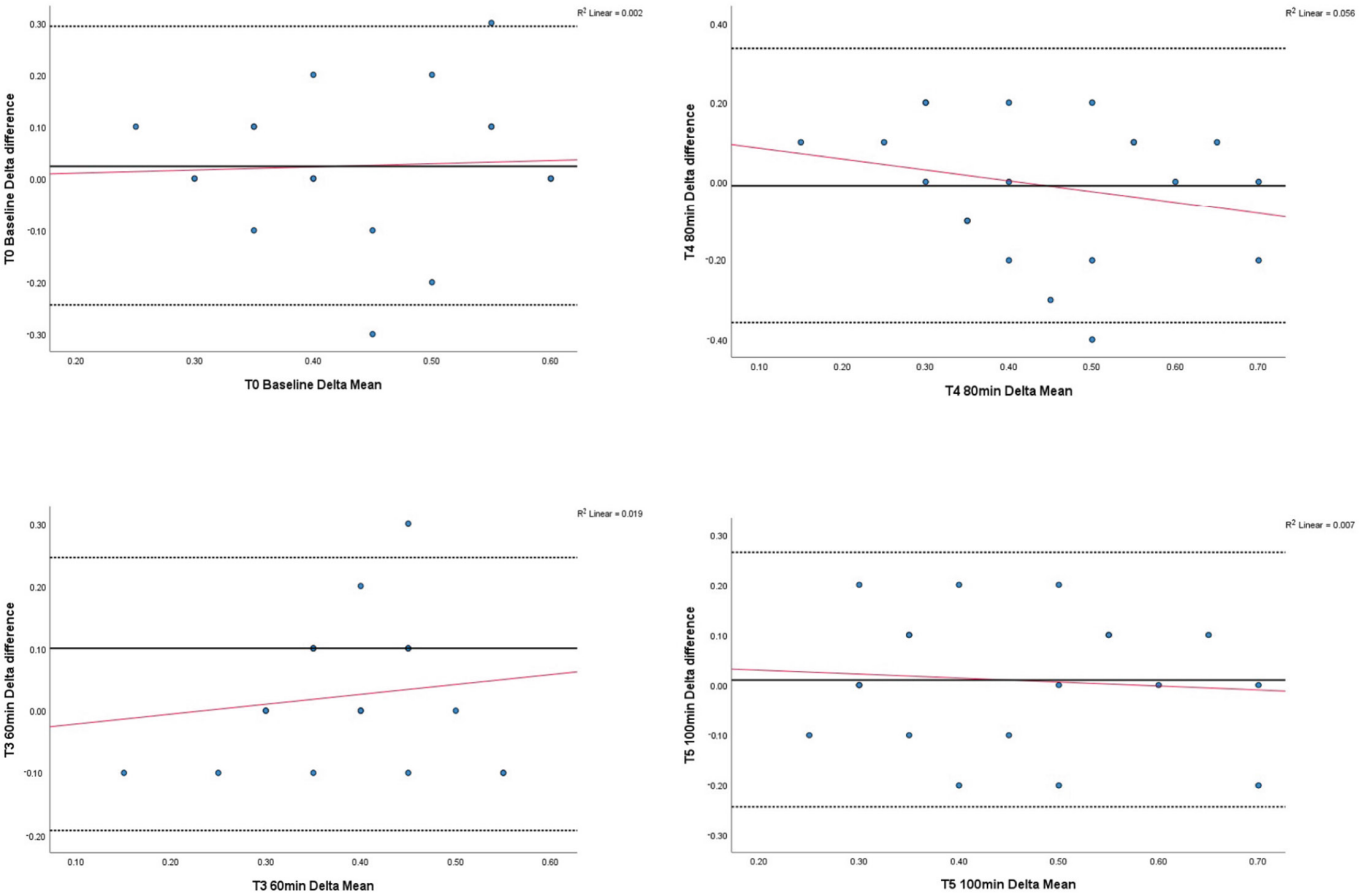
T0, T3, T4, T5 Bland Altman plots: Sacral SEM delta levels of agreement and proportional bias (paired SEM 200 and Provisio®) during 120 min of 60° HOBE (*n* = 20).

A linear regression test for proportional bias (Table 2 and Figure 3) showed a statistically non‐significant *t* value close to zero at baseline (T0), 60‐ (T3), 80‐ (T4) and 100 min (T5), confirming limited differences, or proportional bias, between the SEM 200 and Provisio® sacral SEM delta measurements. Coupled with the large *t* value, visual inspection of the 60 min (T3) Bland Altman plot (Figure 3) suggests some proportional bias is present.

**TABLE 2:** Linear regression test for proportional bias: Sacral SEM delta (paired SEM 200 and Provisio®) during prolonged 60° HOBE (*n* = 20).

	Beta	*t* value	*p* value
Baseline (T0)	0.061	0.205^a^	0.840
60 min (T3)	0.159	0.593^a^	0.560
80 min (T4)	−0.275	−1.030^a^	0.361
100 min (T5)	−0.079	−0.368^a^	0.718

^a^ *t* value close to zero.

## 6 DISCUSSION

This study examined the level of agreement between the SEM 200 and Provisio® devices in measuring sacral SEM delta measurements in healthy adults during 120 min of prolonged 60° HOBE. Our findings contribute to the small body of evidence on these devices (Clendenin et al., 2015; Peko & Gefen, 2019, 2020) and extends our understanding of the device performance in a clinical situation where the sacrum is exposed to prolonged and unrelieved external pressure.

Our study found varying levels of agreement between the two devices over the seven data collection timepoints (T0‐6). There was a good level of agreement in 4 of the 7 (57%) sacral SEM delta measurements between the devices at baseline (T0) and following 60‐ (T3), 80‐ (T4) and 100 min (T5) of prolonged 60° HOBE, with a mean difference between the SEM 200 and Provisio® measurements of 0.025, 0.025, −0.01 and 0.01, respectively. Our findings partially support the laboratory results reported by Peko and Gefen (2019, 2020) who found a good level of agreement in all of their SEM delta measurements in their phantom heel, cheek and chin models when measured by the SEM 200 and Provisio® devices. However, it is difficult to accurately compare our findings because Peko and Gefen (2019, 2020) investigated the sensitivity of the devices in detecting changes in fluid level and their subsequent level of agreement using 0.7 cm thick baby diapers to represent human soft tissue, while our measurements on healthy human sacral tissue means the biophysical responses of the cells and tissues to the external prolonged pressure (Magno et al., 2015) likely contribute to our different findings. Similar to Peko and Gefen (2019, 2020), where a good level of agreement existed, we also found 95% of the sacral SEM delta measurement differences fell within the 95% confidence limits of agreement (Figure 3). Thus, the 5% of datapoints outside of these bounds would be deemed insignificant in the clinical setting. One other study also examined the level of agreement between three SEM 200 devices in healthy human tissue (Clendenin et al., 2015), but the lack of comparison to the Provisio® device means the findings cannot be compared. So, further clinical research on human tissue is needed to determine the level of device agreement in various clinical situations and body positions, which will help to confirm the interchangeability of the SEM 200 and Provisio® devices in clinical practice.

Managing some medical and surgical conditions or preventing complications such as pneumonia require prolonged periods of HOBE (Güner & Kutlutürkan, 2022) which increases sacral tissue loading (Lustig et al., 2020). At three time points, no level of agreement was observed between the two devices following 20‐ (T1), 40‐ (T2) and 120 min (T6) of prolonged 60° HOBE, with a mean difference between the SEM 200 and Provisio® measurements of 0.08, 0.07 and 0.07 respectively. Still, the mean sacral SEM delta measurements at these three timepoints were <0.6 (0.4–0.5), meaning both devices were comparable in terms of their measurements and agreed our healthy volunteers had a lower risk of PI (Bruin Biometrics, n.d.). It is well documented that PI can develop following 1–6 hours of prolonged pressure (European Pressure Ulcer Advisory Panel et al., 2019; Gefen, 2008), with this risk increasing with a HOBE of greater than 30° (European Pressure Ulcer Advisory Panel et al., 2019; Lustig et al., 2020). As such, the early detection of increasing PI risk is paramount (European Pressure Ulcer Advisory Panel et al., 2019), and it would seem the SEM 200 and Provisio® device demonstrate concordance throughout prolonged 60° HOBE. To ensure safe patient care, clinicians need to have confidence that the objective patient data they gather using point‐of‐care technology is reliable and accurate (Bryant et al., 2021). The Provisio® has only been on the market for a few years (Bruin Biometrics, n.d.), with the majority of published clinical evidence based on the SEM 200 (Bone et al., 2022). While the Provisio® has superseded the SEM 200, both devices are currently used in clinical practice, hence more research is needed to compare the devices in a range of clinical situations and patient characteristics such as a range of skin tones.

### 6.1 Limitations and strengths

We acknowledge our small sample of healthy volunteers is not representative of hospitalised medical and surgical patients at risk of PI however, prolonged 60° HOBE is a frequent strategy used in clinical practice. As such, our findings on the level of agreement between the SEM 200 and Provisio® devices in this situation provides important baseline data and confirms the devices are interchangeable in clinical practice.

### 6.2 Recommendations for further research

Our work provides the impetus for further research in hospitalised patient populations to see if results differ. Extending this to include populations with a range of skin tones is also recommended.

## 7 CONCLUSIONS

While the SEM 200 and the Provisio® devices did not achieve the same level of agreement across all seven timepoints, they did determine the same level of PI risk in healthy adults. HOBE is often used clinically to manage medical or surgical conditions, so identifying early PI risk is essential. It seems the objective data gathered using either device could prompt clinicians to implement timely PI prevention strategies which would contribute to improved patient care quality and safety however, more research on both devices is needed to confirm their interchangeability.

## AUTHOR CONTRIBUTIONS

Made substantial contributions to conception and design, or acquisition of data, or analysis and interpretation of data: SL, MB, RW, LT, BG. Involved in drafting the manuscript or revising it critically for important intellectual content: SL, MB, RW, LT, BG. Given final approval of the version to be published. Each author should have participated sufficiently in the work to take public responsibility for appropriate portions of the content: SL, MB, RW, LT, BG. Agreed to be accountable for all aspects of the work in ensuring that questions related to the accuracy or integrity of any part of the work are appropriately investigated and resolved: SL.

## FUNDING INFORMATION

This research received no specific grant from any funding agency in the public, commercial, or not‐for‐profit sectors.

## CONFLICT OF INTEREST STATEMENT

None to declare.

## STATISTICAL ANALYSIS

The authors have checked to make sure that our submission conforms as applicable to the Journal's statistical guidelines. Professor Thalib is a statistician on the author team and checked the statistical analysis presented in the manuscript. The authors affirm that the methods used in the data analyses are suitably applied to their data within their study design and context, and the statistical findings have been implemented and interpreted correctly. The authors agree to take responsibility for ensuring that the choice of statistical approach is appropriate and is conducted and interpreted correctly as a condition to submit to the Journal.

## ETHICS STATEMENT

Ethics approval was obtained from Griffith University Human Research Ethics Committee (GU: 2021/515).

## Data Availability

Research data for this article: Due to the sensitive nature of the study questions, participants were assured raw data would remain confidential and would not be shared.
